# Correction: An approach to the permeation mechanism of learning transfer and teaching strategy in physical education based on complex network

**DOI:** 10.1371/journal.pone.0306650

**Published:** 2024-07-01

**Authors:** The PLOS ONE Staff

The affiliation for the fourth author is incorrect. Yi Zhao is not affiliated with #4 but with #5: The School of Economics and Management, Hebei University of Technology, Tianjin, China.

The publisher apologizes for the error.
