# Supplementary material for: An approach to the permeation mechanism of learning transfer and teaching strategy in physical education based on complex network
Source: PLoS One. 2021 Jan 6;16(1):e0243906. doi: 10.1371/journal.pone.0243906 (PMC7787532; doi:10.1371/journal.pone.0243906)
Supplement: S1 File — (DOCX) [file pone.0243906.s001.docx]

Data Availability Statement

1. A description of the data sets

1. 1Data acquisition method

In this paper, we use Python software to crawl all students' scores under the internal general database of East China Normal University (2007-2008 academic year to 2019-2020 academic year). A total of 78337 data are obtained, including Student ID, student name, student gender, major, course, course, course type, year, and student score. The website of Public Database East China Normal University (ECNU) is <https://portal2020.ecnu.edu.cn/mydashboard>.

1.2 Data preprocessing

(1) Data screening.

We selected the two majors with the largest number of students through data screening. According to the data, sports training and physical education are the two majors with the largest number of students, with 24789 pieces of data and 39933 pieces of data respectively. To unify the year, the students' grades from 2009-2010 to 2019-2020 academic years are selected for both majors.

(2) Data processing.

By Python, the professional basic compulsory courses of the two majors are sorted in descending order according to the number of students in the courses. The top 8 professional basic compulsory courses with the largest number of students in the two professional courses are selected, and 7 of them are the same courses. After sorting out, the students' academic records of the two majors are obtained, that is, each row represents the performance of each student, and each column represents the score of each student in each course. After eliminating the sample of students whose course scores do not exist, 218 sample sizes of students' scores of sports training and 244 sample sizes of physical education majors are obtained. The basic statistical analysis of the average score, standard deviation and variance of seven courses of the two majors and the construction of a complex network diagram can be carried out.

1.3 Data grouping process

(1) Group by year

Considering the different courses taken by students in each academic year, the sample size between each group of years, and the problem that the number of courses will decrease with the number of years grouped, we can divide the year into two groups: 2009-2016 (sample size: 396) and 2017-2019 (sample size: 39). Due to professional adjustment and other issues, some years in the two groups of years have no data, but it does not affect the research results. The courses taken by the students in the two groups were "Sports Physiology, Sports Anatomy, Sports Statistics, Sports Psychology，Sports Introduction，Sports Sociology，Sports Research Methods".

(2) Group by the average score

When we do the multi-layer network chart, we also take the average score as the dividing line, and sort out that the sample size higher than average score is 94, and the sample size lower than average score is 91; the sample size higher than average score is 116, and the sample size lower than average score is 110.

(3) Group by gender

The sample size of male students is 120, that of girls is 98; that of boys in physical education is 199, and that of girls is 45.

1.4 Document description

Two groups of data are stored in the file named "matlab": "data"_ danceng” and“data_ duoceng”.

（1）data_ danceng: construct the relevant calculation data of single-layer network diagram, construct the "seven professional courses link network diagram of physical education major", "2009-2016 curriculum link network diagram" and "2017-2019 curriculum network link diagram", also including the "node degree statistics" of these three groups of network graphs under different threshold values, and select the "between any two courses" under the determined threshold Short path length, average shortest path length, betweenness of each course node, clustering coefficient of each course node, etc.

（2）data_ duocheng: the relevant calculation data of multi-layer network chart is constructed, including "the link network diagram of eight courses of two sports majors", "the link network diagram of eight courses of two sports majors with higher or lower average scores" and "the link network diagram of eight courses of two sports majors under different genders", which also includes the "sports training major" of the five groups of network graphs The second characteristic value of the network should be transferred to physical education major.

1. The reason for the restriction

PLOS only allows data to be available upon request if there are legal or ethical restrictions on sharing data publicly. Our data are all from <https://portal2020.ecnu.edu.cn/mydashboard>. But only the teacher has access to this website to obtain the results of the students he/she teaches. In these data, there is some students' personal information, including student ID, gender, etc. Therefore, The College of Physical Education & Health, East China Normal University does not recommend that it be publicly visited. Although we have hidden some of his privacy information in the process of research, there are still some of them that may involve the privacy of students, so we do not want to make these data public. If there is a third-party organization or individual research for non-profit purposes, our corresponding author can be contacted to obtain some data, and we will offer the data according to the research needs.

The restricting institutional body: The College of Physical Education & Health of East China Normal University.

Non-author contact information: Zhongyin Zhang, [zyzhang@tyxx.ecnu.edu.cn](mailto:zyzhang@tyxx.ecnu.edu.cn).
