# Supplementary material for: An approach to the permeation mechanism of learning transfer and teaching strategy in physical education based on complex network
Source: PLoS One. 2021 Jan 6;16(1):e0243906. doi: 10.1371/journal.pone.0243906 (PMC7787532; doi:10.1371/journal.pone.0243906)
Supplement: Attachment — Submitted filename: Response to Reviewers.doc [file pone.0243906.s003.doc]

Dear Dr.Carlos (Academic Editor)and reviewers:

We appreciate you very much for your constructive comments and suggestions on our manuscript entitled”An approach to the Permeation Mechanism of Learning Transfer and Teaching Strategy in Physical Education Based on Complex Network”. We have studied all of comments carefully. According to the suggestions, we make the following explanations on the explanation and modification of the relevant contents of the paper.

1. Formatting issues, especially with references.

Thank you for your valuable suggestions. We carefully browse the articles and refer to the relevant formats of other articles in PLOS One. We have made a corrigendum to 22	references in this paper, and the relevant information of each reference has been consulted and corrected item by item The parts with problems have been corrected according to	the format requirements of PLOS One, and all of them have been corrected in the paper.

2. Unnecessarily having a large number of decimal places, when there is no idea about precision and error.

Thank you for your valuable comments. To solve this problem, we searched the PLOS One official website for papers with similar topics and found that most of the effective digits are two digits after the decimal point. We carefully refer to the opinions of the evaluation, and now we change the values in the tables such as “The shortest path length between any two courses” and “The clustering coefficient of each course node” from “four decimal places” to “two decimal places”. Because there are too many decimal places and small values in the table of “The second smallest eigenvalue of the transfer network from major 1 to 2”, if the values are kept to two decimal places, no obvious difference can be found. Therefore, the numerical value is changed into the form of scientific counting method. For example, the second small eigenvalue of “sports physiology” is changed from “0.1699” to “1.70e-1” in Table 16, which can more accurately reflect the difference between the values. I hope you will check again. If there are still some deficiencies, please criticize and correct them.

3. Please include the details in the figure captions. Otherwise some captions appear to be repeated. Have a look at other articles from Plos.

First of all, I would like to thank you for your valuable suggestions. For the suggestions given, we refer to other articles of PLOS One. In order to make the graphic title more clear, the following changes are made to some graphic titles:

(1) Single layer network diagram (page 9-18)

We changed the title of **Figure 4** from "**The linked network of 7 courses for two sports majors**" to "**The linked network of 7 courses of sports major from 2009 to 2019**", and the title of **Figure 5** was changed from "**The** **course link network diagram**" to "**The linked network of 7courses of sports major from 2009 to 2016**". The title of Figure 6 is changed from "The **Course link network diagram**" to "**The linked network of 7courses of sports major from 2017 to 2019**".

(2) Multi layer network diagram part (article page 21-29)

We changed the title of Figure 8 from " **Link network of eight courses for two physical education majors**" to "**The linked network of 8 courses with higher than average scores of students majoring in Physical Education**". The title of Figure 9 was changed from "**Link network of eight courses for two physical education majors**" to "**The linked network of 8 courses with lower than average scores of students majoring in Physical Education**", and the title of Figure 10 was changed from "**Link network of eight courses for two physical education majors**" to “**The linked network of the scores of 8 courses for male students majoring in Physical Education**”.

The title of Figure 11 is changed from "**Link network of eight courses for two physical education majors**" to "**The linked network of the scores of 8 courses for female students majoring in Physical Education**".

In addition, in order to make the graphic information display more comprehensive, we also make a more detailed description of the selection of graphics data and related information. For example:

(1) The description content of Figure 4 is “Figure 4 shows that after calculating the correlation of seven professional basic courses of 462 students majoring in sports training and physical education in East China Normal University, the different course linked network diagrams are got through dynamically changing the threshold K. The seven courses are respectively sports physiology, sports anatomy, sports statistics, sports psychology, sports introduction,Sports Sociology, Sports Sociology. The threshold value of figure a is 0.3, that of figure b is 0.4, and that of figure c is 0.5. ”

(2) The description content of Figure 7 is "Figure 7 is a multiple network of learning transfer for sports training and physical education majors of East China Normal University through the same curriculum coupled with different professional curriculum networks. Among them, there are 7 courses of the same two majors, which are sports physiology, sports anatomy, sports statistics, sports psychology, sports introduction, sports sociology, sports research methods, and different courses are "Sports Training Major - Sports Training" and "Physical Education Major - sports biochemistry". 218 samples of sports training and 244 samples of physical education were selected.” Please see the paper for other details.

4. Permeation (appears in the title) would have been a better word than infiltration. Authors should rethink.

Thank you for your suggestions. We read the article carefully and changed the term “infiltration” to “permeation”.In addition, due to the change of the author's job, the affiliated institution of the author in the article has been modified.

Finally, we would like to express our sincere thanks to all of you for your carefulness and seriousness!We look forward to receiving your feedback as soon as possible.

Kind regards,

Zhao Yi

Fudan University
